# Synthesis and characterization of aluminosilicate and zinc silicate from sugarcane bagasse fly ash for adsorption of aflatoxin B1

**DOI:** 10.1038/s41598-024-65158-2

**Published:** 2024-06-24

**Authors:** Chalida Niamnuy, Sirada Sungsinchai, Prapaporn Jarernsamrit, Sakamon Devahastin, Metta Chareonpanich

**Affiliations:** 1 Department of Chemical Engineering, Faculty of Engineering, Kasetsart University, 50 Ngam Wong Wan Road, Chatuchak, Bangkok, 10900 Thailand; 2 Center for Advanced Studies in Nanotechnology and Its Applications in Chemical, Food and Agricultural Industries, Kasetsart University, 50 Ngam Wong Wan Road, Chatuchak, Bangkok, 10900 Thailand; 3 School of Food Industry, King Mongkut’s Institute of Technology Ladkrabang, Bangkok, 10520 Thailand; 4 Advanced Food Processing Research Laboratory, Department of Food Engineering, Faculty of Engineering, King Mongkut’s University of Technology Thonburi, 126 Pracha U-Tid Road, Tungkru, Bangkok, 10140 Thailand; 5 The Academy of Science, The Royal Society of Thailand, Dusit, Bangkok, 10300 Thailand

**Keywords:** Electrostatic interaction, Metal silicate, Surface acidic site, Waste utilization, Zeta potential, Chemical engineering, Structural properties

## Abstract

Sugarcane bagasse fly ash, a residual product resulting from the incineration of biomass to generate power and steam, is rich in SiO_2_. Sodium silicate is a fundamental material for synthesizing highly porous silica-based adsorbents to serve circular practices. Aflatoxin B1 (AFB1), a significant contaminant in animal feeds, necessitates the integration of adsorbents, crucial for reducing aflatoxin concentrations during the digestive process of animals. This research aimed to synthesize aluminosilicate and zinc silicate derived from sodium silicate based on sugarcane bagasse fly ash, each characterized by a varied molar ratio of aluminum (Al) to silicon (Si) and zinc (Zn) to silicon (Si), respectively. The primary focus of this study was to evaluate their respective capacities for adsorbing AFB1. It was revealed that aluminosilicate exhibited notably superior AFB1 adsorption capabilities compared to zinc silicate and silica. Furthermore, the adsorption efficacy increased with higher molar ratios of Al:Si for aluminosilicate and Zn:Si for zinc silicate. The N_2_ confirmed AFB1 adsorption within the pores of the adsorbent. In particular, the aluminosilicate variant with a molar ratio of 0.08 (Al:Si) showcased the most substantial AFB1 adsorption capacity, registering at 88.25% after an in vitro intestinal phase. The adsorption ability is directly correlated with the presence of surface acidic sites and negatively charged surfaces. Notably, the kinetics of the adsorption process were best elucidated through the application of the pseudo-second-order model, effectively describing the behavior of both aluminosilicate and zinc silicate in adsorbing AFB1.

## Introduction

Mycotoxins, produced by certain fungi, pose a significant health risk to both humans and livestock. Aflatoxin stands out as one of the most concerning mycotoxins^1^. Among the various types of aflatoxins, aflatoxin B1 (AFB1) is notable for its toxicity and widespread presence in animal feeds,it constitutes a significant area of concern^2^. To mitigate AFB1 contamination in both human foods and animal feeds, various commercial adsorbents are frequently employed, including activated carbon, hydrated sodium calcium aluminosilicate, dietary clay, zeolites, bentonite clay^3^, and silica. These materials are selected for their significant pore volumes and extensive surface areas, facilitating effective AFB1 adsorption. However, the utilization of certain adsorbents presents drawbacks. For example, zeolites and hydrated sodium calcium aluminosilicate may require high energy consumption during synthesis and could potentially release toxic components, such as heavy metals or dioxins^4^. Activated carbon, while widely used due to its cost-effectiveness, features fine pore characteristics that might hinder the absorption of larger AFB1 molecules^5^. Additionally, although several clay types show promising abilities in AFB1 adsorption, there is a potential risk of contamination with hazardous metals such as arsenic, mercury, and lead. Silica, considered as an alternative for AFB1 adsorption, exhibits limited effectiveness in addressing this issue. Consequently, the selection of an appropriate adsorbent material continues to pose a persistent challenge in combating AFB1 contamination.

Sugarcane bagasse fly ash is a byproduct generated in enormous quantities during the production of sugar and ethanol, where bagasse serves as a primary fuel for generating power and steam. This fly ash results from the incomplete combustion of bagasse^6^, posing a significant environmental threat and encouraging limitations for its direct utilization^7,8^. The need to transform this agro-industrial waste into valuable resources is, therefore, very important. The composition of bagasse fly ash consists mainly of unburned carbon, SiO_2,_ and Al_2_O_3_. Various researchers have reported converting this fly ash into useful porous silica using an environmentally sustainable method^6^. These porous silica-based materials offer a wide range of applications in various industries. They serve as catalysts, catalyst support materials, additives in cement and concrete, as well as adsorbents^9,10^.

The silica-based adsorbent has garnered considerable attention due to its attributes, including higher surface area, ordered porosity, narrow pore size distribution, high thermal stability, and ease of synthesis. Among these characteristics, aluminosilicate, a derivative of silica, has demonstrated remarkable adsorption capabilities across various applications. The bonding of aluminum and silicon within aluminosilicate enhances the surface acidic sites compared to pure silica. Numerous studies have underscored the high effectiveness of aluminosilicate in adsorbing mycotoxins^11^. However, there remains a lack of reports on both the synthesis process and the AFB1 adsorption capacity of aluminosilicate derived from sugarcane bagasse fly ash. Among metal silicates, zinc silicate emerges as a cost-effective material used as an adsorbent for removing dye from water sources^12^. Research by Nones et al.^13^ indicated that bentonite modified with zinc exhibited an enhanced AFB1 adsorption capacity compared to natural bentonite, attributed to structural changes resulting from modifications. Nevertheless, there is very limited research reported on the AFB1 adsorption capacity of zinc silicate. Moreover, studies have not reported evidence of toxicity associated with either aluminum or zinc following oral intake at low doses^14^. In this study, the synthesis of aluminosilicate and zinc silicate was conducted with a focus on minimal consumption of aluminum and zinc, employing a basic one-pot method for synthesis, aligning with circular practices. This information demonstrates the potential as a guiding principle for the future development of silica-based adsorbents, especially those derived from agricultural wastes such as sugarcane bagasse fly ash.

Based on the arguments, this study aimed to explore the synthesis of aluminosilicate and zinc silicate using sugarcane bagasse fly ash as a silica source. Additionally, the investigation focused on evaluating specific physicochemical properties of the aluminosilicate and zinc silicate adsorbents, examining their efficacy in adsorbing AFB1 during the in vitro intestinal phase.

## Experimental

### Preparation of sodium silicate from bagasse fly ash

The procedure for synthesizing sodium silicate from bagasse fly ash was executed according to the methodology outlined by Ruengrung et al.^5^ and Sungsinchai et al.^15^. Initially, 18.648 g of bagasse fly ash, containing 74.23% silicon dioxide (SiO_2_), was amalgamated with 22.601 g of sodium carbonate (Na_2_CO_3_, sourced from Kemaus, NSW, Australia), and underwent thorough homogenization for 30 min. Subsequently, the resultant composite underwent calcination at a temperature of 850 °C for 1 h, employing a heating rate of 10 °C/min, thereby yielding a blue-hued solid precipitate. Following this, deionized (DI) water was introduced to the precipitate, followed by centrifugation at a rate of 8900 rpm for 10 min, resulting in the separation of a green-colored supernatant solution. This solution was subjected to desiccation at a temperature of 150 °C for 24 h, subsequently followed by a further round of calcination at 850 °C for an additional hour, culminating in the production of sodium silicate.

### Preparation of porous silica (SiO2)

The methodology for obtaining SiO_2_ was following the protocol established by Sungsinchai et al.^15^. The resultant sodium silicate was mixed with deionized (DI) water to formulate a sodium silicate solution. Subsequently, this solution was incrementally introduced into an acetic acid solution (2% v/v) under agitation. Continuous stirring was sustained throughout the process, and the pH of the solution was adjusted to 6 by adding 1 M hydrochloric acid (HCl) and 1 M sodium hydroxide (NaOH) solutions into the mixture at 40 °C. After 6 h, the resultant gel was transposed into a Teflon-lined autoclave, wherein it underwent aging at a temperature of 100 °C for 24 h. Thereafter, the gel underwent sequential processes of filtration, rinsing with DI water, and desiccation at 100 °C for 24 h. The dried sample was subsequently subjected to calcination at a temperature of 600 °C for 4 h, employing a heating rate of 2 °C/min. Following calcination, the sample was ground to a particle size ranging between 53 and 106 µm and subsequently preserved at ambient conditions for subsequent analyses.

### Preparation of aluminosilicate

Aluminosilicates were prepared with varying molar ratios of aluminum (Al) to silicon (Si), denoted as AS(0.02), AS(0.04), and AS(0.08), and corresponding to ratios of 0.02, 0.04, and 0.08, respectively. Initially, a specific quantity of sodium silicate was added to DI water to create a solution. This sodium silicate solution was slowly dropped into a 2% v/v acetic acid solution while stirring. The mixture was continuously stirred at 40 °C while gradually adding 1 M HCl and 1 M NaOH solutions to achieve a pH of 6. Concurrently, an appropriate quantity of aluminum nitrate nonahydrate (Al(NO_3_)_3_·9H_2_O) solution necessary to achieve the desired ratio was prepared. Subsequently, the solution of aluminum nitrate nonahydrate was slowly mixed with sodium silicate solution under stirring at 40 °C. The pH of the solution was carefully maintained at 6.0. After stirring for 6 h, the mixture was aged in a Teflon-lined autoclave at 100 °C for 24 h. Following this, it underwent filtration, rinsing with DI water, and was then dried at 100 °C for 24 h. The resulting solid was subjected to calcination at 600 °C for 4 h, with a gradual heating rate of 2 °C/min. Subsequently, the sample was sieved to achieve particle sizes ranging from 53 to 106 µm and stored at room temperature for further analysis.

### Preparation of zinc silicate

The zinc silicates denoted as ZS(0.02), ZS(0.04), and ZS(0.08) with respective zinc (Zn) to silicon (Si) molar ratios of 0.02, 0.04, and 0.08, were synthesized. Initially, a specific quantity of sodium silicate was dissolved in DI water. Subsequently, this sodium silicate solution was gradually added to a 2% v/v acetic acid solution while stirring. Continuous stirring at 40 °C was maintained while dropping 1 M HCl and 1 M NaOH solutions to adjust the pH to 6. Concurrently, an appropriate quantity of zinc nitrate hexahydrate (Zn(NO_3_)_2_·6H_2_O) solution, required to achieve the desired ratio, was prepared. The zinc nitrate hexahydrate solution was then slowly combined with the sodium silicate solution, with continuous stirring maintained at 40 °C. The pH of the solution was adjusted to 6.0. After stirring for 6 h, the resulting mixture was aged in a Teflon-lined autoclave at 100 °C for 24 h. Following this, it underwent filtration, rinsing with DI water, and drying at 100 °C for 24 h. The dried sample was then calcined at 600 °C for 4 h, with a heating rate of 2 °C/min. Subsequently, the product was sieved to achieve particle sizes ranging from 53 to 106 µm and stored at room temperature for further analysis.

### Surface properties determination

The surface properties of the porous silica, namely, BET surface area, pore size, and pore volume, were assessed using N_2_ physisorption analysis carried out with Autosorb®-1-C equipment (Quantachrome Instruments, Boynton Beach, FL). Both BET and BJH methods were employed at a temperature of − 196 °C. Before analysis, the sample underwent degassing at 200 °C^16^.

### X-ray diffraction

The sample underwent X-ray diffraction (XRD) analysis using a Bruker D8 advance diffractometer (Bruker AXS GmbH, Karlsruhe, Germany). CuKα radiation (λ = 1.5406 Å) was employed at 40 kV and 40 mA. The data collection utilized a scan rate of 0.02°/0.5 s. The scan covered a wide 2θ range of 5°–80°^17^.

### Fourier transform infrared spectroscopy

Fourier-transform infrared (FTIR) spectroscopy was employed to identify the functional groups within the sample. The analysis was performed using a PerkinElmer Spectrum One spectrometer (PerkinElmer, Shelton, CT, USA) across a wide infrared range spanning 4000–400 cm^−1^.

### X-ray photoelectron spectroscopy

X-ray photoelectron spectroscopy (XPS) was utilized to investigate the elemental composition at the surface of each adsorbent sample. A Kratos Axis Ultra DLD spectrometer (Kratos, San Diego, CA, USA) was utilized for this analysis. To ensure binding energy accuracy, the XPS peaks were calibrated by setting the C1s and O1s peaks to 284.5 eV and 531 eV, respectively.

### Morphological assessment

A field-emission scanning electron microscope (FE-SEM) (JEOL, JSM-7610F, Tokyo, Japan) was utilized to monitor the morphology of the samples. The samples were coated with Platinum (Pt) at 1.00 kV and magnified to 15,000×. Additionally, transmission electron microscopy (FE-TEM) (JEOL, JEM-3100F, Tokyo, Japan) was employed with an accelerating voltage of 300 kV to assess the microstructure of the samples.

### Temperature-programmed desorption using ammonia as probe molecule (NH3-TPD)

The temperature-programmed desorption apparatus (Thermo Scientific, TPDRO 1100, Waltham, MA) was utilized to examine the acidic properties of porous silica. Initially, 0.2 g of the sample underwent pretreatment by passing helium (He) at a flow rate of 30 mL/min, with a heating rate of 10 °C/min until reaching 400 °C, which was maintained for 60 min. The sample was then cooled to 100 °C. After that, a 10% v/v NH_3_ (with He as a balance gas) was introduced to the sample for 1 h at a flow rate of 30 mL/min. Following the NH_3_ flow disconnection, the sample was flushed with He at a rate of 30 mL/min for 1 h to remove physisorbed NH_3_. The NH_3_ desorption analysis was conducted by flowing He (30 mL/min) while heating the sample from 100 to 600 °C at a rate of 10 °C/min. The intensity of the acid sites was evaluated by monitoring the weight loss result of NH_3_ desorption^15^.

### Zeta potential determination

Zeta potential was measured with a Zetasizer (Malvern Panalytical, Zetasizer Nano ZSP, Worcestershire, UK). Measurements were taken at pH 2.5 and 6.5 to mimic the conditions during in vitro digestion in the gastric and intestinal phases, respectively.

### UV–Vis spectrophotometry

The UV–Vis spectra of the AFB1 solution at various periods of adsorption were evaluated using a double-beam UV–Vis spectrophotometer (PG Instruments, T92+, Lutterworth, UK).

### In vitro Aflatoxin B1 (AFB1) adsorption

#### Standards preparation

AFB1 standards for HPLC analysis were arranged following the method outlined by Sungsinchai et al.^18^ at concentrations of 0, 0.875, 1.75, and 3.5 µg/mL.

#### Preparation of artificial gastric and intestinal juices

The preparation of artificial gastric juice (AGJ) and artificial intestinal juice (AIJ) followed the methodologies described by Tso et al.^19^ and Sungsinchai et al.^15^. Initially, a solution was formed by dissolving 1 g of NaCl and 1.6 g of pepsin in distilled water. Subsequently, 2.5 mL of 36.5% HCl was added, and the resulting solution was diluted to a final volume of 500 mL with distilled water to create AGJ. The pH of AGJ was then adjusted to 2.5 using 0.1 M NaOH. For the preparation of AIJ, 3.4 g of KH_2_PO_4_ was dissolved in 250 mL of distilled water, and the pH was adjusted to 6.8 using 0.1 M NaOH. In a separate container, 5 g of trypsin was dissolved in distilled water and combined with the KH_2_PO_4_ solution. Subsequently, 1.5 g of porcine bile salt was added to this mixture, and the volume was adjusted to 500 mL with distilled water to obtain AIJ. The pH of AIJ was then adjusted to 6.5 using NaOH solution (0.1 M) and HCl solution (36.5%). The prepared AGJ and AIJ were stored at 4 °C for subsequent use.

#### In vitro digestion and chromatographic conditions of AFB1

The in vitro digestion process was conducted following the methodology proposed by Tso et al.^19^ and Sungsinchai et al.^15^. Initially, 1 mL of AFB1 solution (3.5 µg/mL) was added to 21 mL of AGJ, followed by the addition of 3.5 mg of each synthesized adsorbent. The mixture underwent incubation at 40 °C under shaking at 150 rpm for intervals of 0, 1, 3, and 5 h. After the 5-h digestion period, 20 mL of AIJ was added to the mixture and continuously incubated for durations of 0.5, 1, 1.5, and 2 h. Subsequently, the mixture was filtered using Whatman paper No. 3. The resulting extract underwent clean-up and HPLC analysis following the procedures outlined by Sungsinchai et al.^18^. All tests were performed in duplicate and the average values were reported.

To analyze the adsorption kinetic data, adsorption kinetic equations were applied. The adsorption capability is quantified by Eq. (1).

1 $${\text{Adsorption}}\,\,{\text{capability}}\,\,( \%)\,\, = \,\,\frac{{\left( {C_{\it{0}} - C_{t} } \right)}}{{C_{\it{0}} }} \times 100$$

where *C*_o_ is initial concentration of AFB1 (µg/mL), and *C*_t_ is the concentration of AFB1 at the final stage of in vitro digestion (µg/mL).

The adsorption kinetics of AFB1 were analyzed using three models: the pseudo-first-order model, pseudo-second-order model, and intra-particle diffusion model^20^. As previously mentioned, the initial concentration of AFB1 was 3.5 µg/mL. The pseudo-first-order model is commonly used to interpret data obtained from the adsorption of various adsorbates from a solution. This model describes the adsorption rate, which correlates with the unoccupied binding sites on an adsorbent. In contrast, the pseudo-second-order model represents an adsorption kinetic model elucidating the interaction between the functional groups existing on the surface of the adsorbent and the adsorbate, influencing adsorption through chemical bonds. Equations 2 and 3, as provided by Ebelegi et al.^21^, outline the formulations used for the pseudo-first-order and pseudo-second-order models, respectively.

2 $$ {\text{ln}} \left( {{\text{q}}_{{\text{e}}} - {\text{ q}}} \right){\text{ = ln q}}_{{\text{e}}} - {\text{k}}_{{1}} {\text{t}}$$

3 $$\frac{{\text{t}}}{{\text{q}}}{ = }\frac{{\text{t}}}{{{\text{q}}_{{\text{e}}} }}{ + }\frac{{1}}{{{\text{k}}_{{2}} {\text{q}}_{{\text{e}}}^{{2}} }}$$

where *q*_e_ (mg/g), *k*_1_ (1/sec), and *q* (mg/g) represent the quantities of AFB1 adsorbed onto the adsorbent at equilibrium and at any given time during the adsorption process (s), respectively. Additionally, *k*_2_ (g/mg⋅sec) denotes the kinetic rate constant for both the pseudo-first-order and pseudo-second-order models.

4 $${\text{q = k}}_{{\text{p}}} {\text{t}}^{{{0}{\text{.5}}}} {\text{ + C}}$$

where* k*_p_ is the intra-particle diffusion rate constant (mg/g⋅sec^0.5^) and *C* remains a constant for any experiment.

### Static adsorption isotherms and thermodynamic studies

Batch static adsorption experiments were conducted in 100-mL Erlenmeyer flasks. For each experiment, the AFB1 solution was with a volume of 42 mL, and the adsorbent dosage was 3.5 mg dry mass. The contact time for AFB1 adsorption onto adsorbent samples was varied between 0 and 8 h. An effect of the initial concentration of AFB1 solution was assessed by varying it from 0.3 to 0.9 µg/mL in DI water, and the temperature was maintained at 40 °C. The impact of temperature was explored in the range of 30–50 °C at an initial concentration of AFB1 solution of 0.5 µg/mL. Each adsorbed AFB1 solution was analyzed by high-performance liquid chromatography (HPLC) to determine the residual AFB1 content. All tests were performed in duplicate and the average values were reported.

Adsorption isotherms are generally determined to clarify possible interactions between the adsorbent and the adsorbate. These interactions are critical for establishing the optimal adsorption capacity of the adsorbent at a constant temperature. In this study, the equilibrium adsorption data of AFB1 were analyzed using two different adsorption isotherms: the Langmuir and Freundlich isotherms. The Langmuir isotherm equation is commonly used to model monolayer adsorption on a homogeneous adsorbent surface. The linear form of the Langmuir isotherm is calculated as follows^22^:

5 $$\frac{{{\text{C}}_{{\text{e}}} }}{{{\text{q}}_{{\text{e}}} }}{ = }\frac{{1}}{{{\text{bq}}_{{\text{m}}} }}{ + }\frac{{{\text{C}}_{{\text{e}}} }}{{{\text{q}}_{{\text{m}}} }}$$

where *q*_e_ is the equilibrium adsorption capacity (mg/g), *C*_e_ is the equilibrium concentration of adsorbed AFB1 solution (mg/L), *q*_m_ is the maximum adsorption capacity at the saturation of a monolayer (mg/g) and *b* is the Langmuir constant (L/mg).

Freundlich isotherms describe the adsorption of multiple layers on heterogeneous surfaces. The linear form of the Freundlich isotherm was as follows:

6 $${\text{log q}}_{{\text{e}}} {\text{ = log K}}_{{\text{F}}} { + }\frac{{1}}{{\text{n}}}{\text{ log C}}_{{\text{e}}}$$

where *K*_F_ ((mg/g)(L/mg)^1/*n*^) and *n* (dimensionless) are the constants of the Freundlich model.

7 $$ \Delta {\text{G}}^{^\circ } { = } - {\text{RTlnK}}_{{\text{D}}}$$

Thermodynamic parameters are essential for evaluating the orientation and feasibility of physicochemical adsorption reactions. These parameters provide valuable insights into the inherent energy and structural dynamics of the system. The Gibbs free energy change (∆${\text{G}}^{^\circ }$) of the adsorption process is related to the equilibrium constant (*K*_D_) by the classical Van’t Hoff equation as follows^23,24^:

where R is the universal gas constant *(*8.314 J K^−1^ mol^−1^). The equilibrium constant *(K*_D_*)* can be determined by Eq. (8):

8 $${\text{ K}}_{{\text{D}}} { = }\frac{{{\text{q}}_{{\text{e}}} }}{{{\text{C}}_{{\text{e}}} }}$$

The Gibbs free energy change *(*∆${\text{G}}^{^\circ }$, J mol^−1^) is also related to the enthalpy change *(*∆${\text{H}}^{^\circ }$, J mol^−1^*)* and the entropy change *(*∆${\text{S}}^{^\circ }$, J K^−1^ mol^−1^*)* at constant absolute temperature *(*T, K*)* according to the following equation:

9 $$\Delta {\text{G}}^{^\circ } \,{ = }\,\Delta {\text{H}}^{^\circ } \, - \,{\text{T}}\Delta {\text{S}}^{^\circ }$$

The combination of the Eqs. (7) and (9) gets Eq. (10).

10 $${\text{ lnK}}_{{\text{D}}} { = }\frac{{ - \Delta {\text{G}}^{^\circ } }}{{{\text{RT}}}}{ = }\frac{{\Delta {\text{S}}^{^\circ } }}{{\text{R}}} - { }\frac{{\Delta {\text{H}}^{^\circ } }}{{\text{R}}}\frac{{1}}{{\text{T}}}$$

### Reusability evaluation

Approximately 0.10 g of AS(0.08) adsorbent was first contacted with 1200 mL of 0.5 µg/mL of AFB1 solutions. The experiments were carried out in an isothermal water bath shaker with a rotation speed of 150 rpm at 40 °C for 8 h. After the adsorption equilibrium was reached, the adsorbent was directly separated from the solution and immersed in 200 mL of acetate buffer (10 mM, pH 5)^25^. The desorption process was carried out in a shaker at 150 rpm at 30 °C for 8 h. The desorbed adsorbent was dried at 60 °C for 12 h and then used for another adsorption.

### Statistical analysis

The data were analyzed using one-way analysis of variance (ANOVA), and the results are presented as mean values with standard deviations based on two replicates. Tukey’s multiple range test was employed to determine the significance among the mean values at a 95% confidence level. All statistical analyses were performed using SPSS® software, version 22 (SPSS Inc., Chicago, IL, USA).

## Results and discussion

### Surface area and pore size distribution

**Figure 1:**
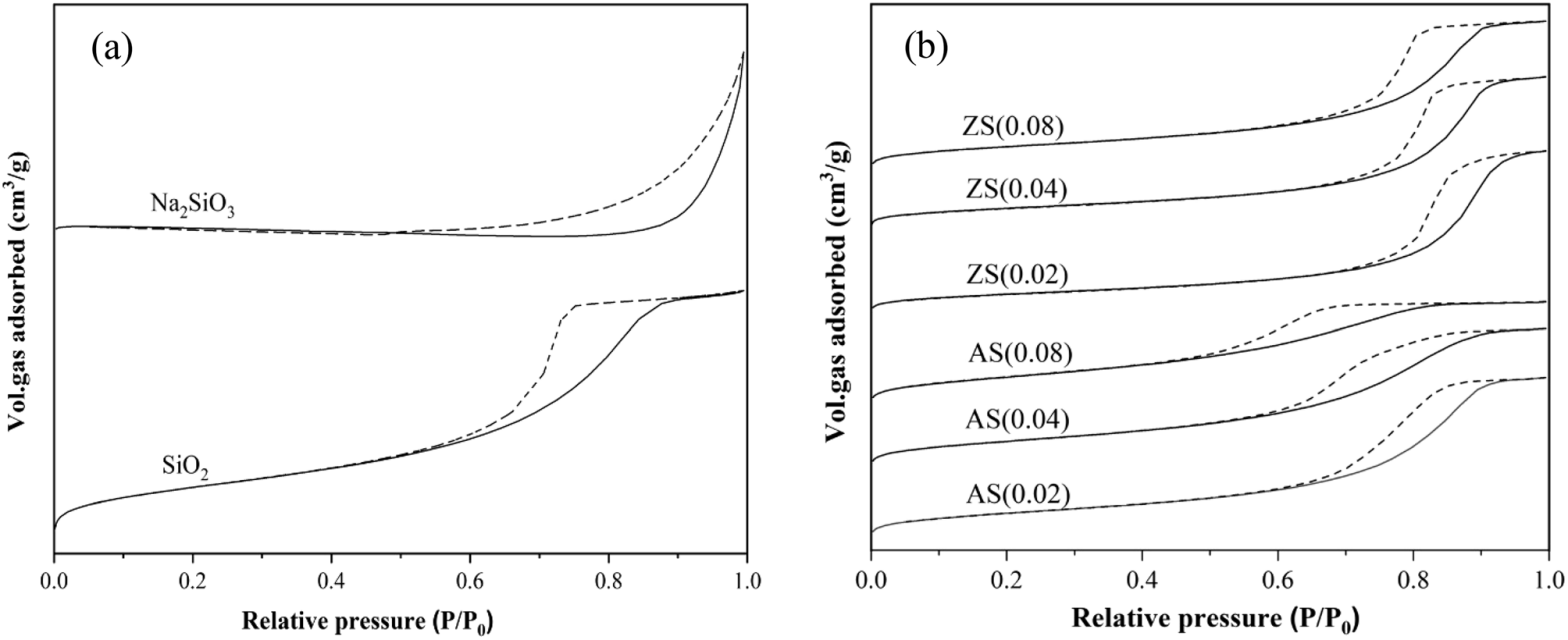
Nitrogen sorption isotherms of (**a**) sodium silicate and silica, (**b**) Aluminosilicate and zinc silicate. Legends:–adsorption - - desorption.

The adsorption–desorption isotherms of porous silica, aluminosilicate, and zinc silicate are illustrated in Fig. 1. Since capillary condensation occurs in mesopores, the desorption path is expected to differ from the adsorption path, resulting in the formation of a hysteresis loop^26^. Porous silica, aluminosilicate, and zinc silicate exhibited type IV isotherms with hysteresis loops, indicating the presence of a mesoporous structure. All aluminosilicate and zinc silicate adsorbents exhibited sinusoidal-pore silica with a monomodal structure. These pore structures were formed by the aggregation of silica and metals such as aluminum or zinc nanoparticles, resulting in an interconnected wormhole structure^17^. The hysteresis loops of porous silica, aluminosilicate, and zinc silicate are identified as H2, implying that their pores have an ink-bottle shape. Liu et al.^27^ suggested that mesopore volume with ink-bottle pores is advantageous for adsorption.

**Figure 2:**
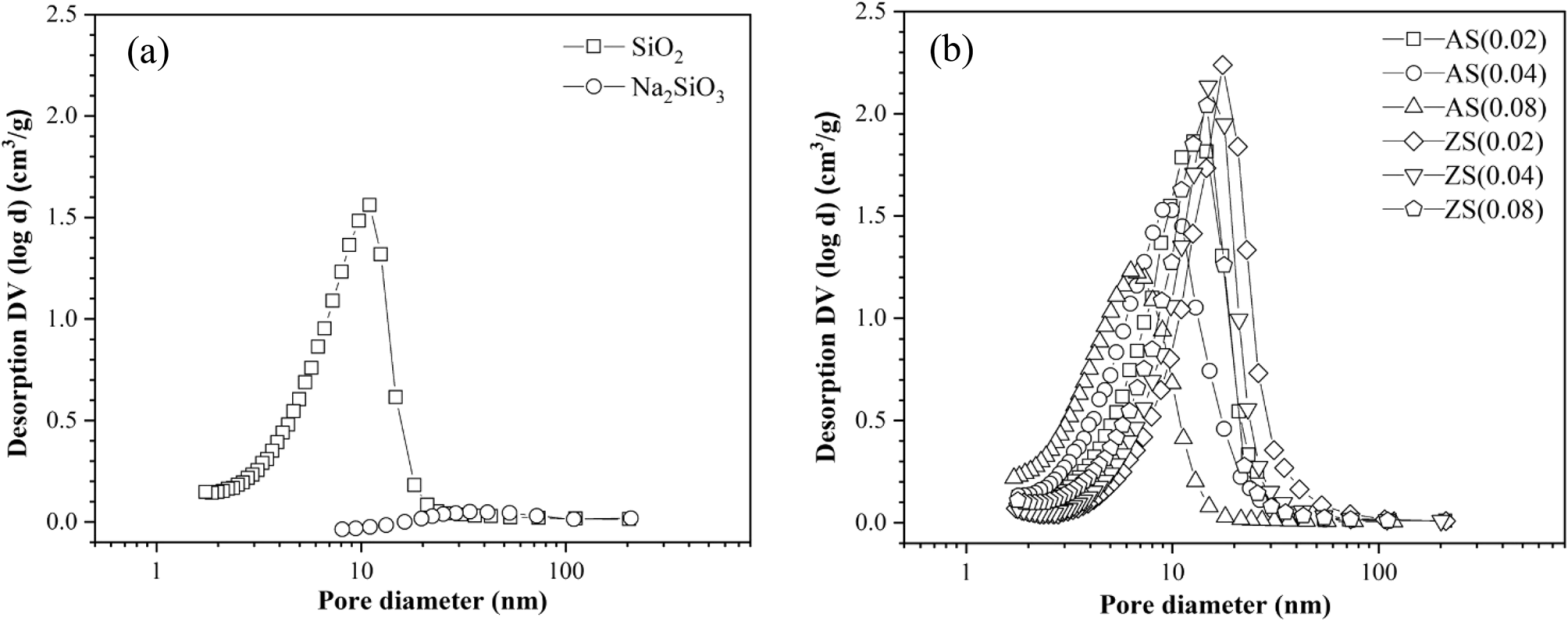
Pore size distribution of (**a**) sodium silicate and silica (**b**) aluminosilicate and zinc silicate.

Figure 2 illustrates the pore size distribution of porous silica, aluminosilicate, and zinc silicate. All samples of porous silica, aluminosilicate, and zinc silicate exhibited narrow monomodal pore sizes, indicating a uniform pore size and structure^17^. These findings were consistent with the average pore diameters presented in Table 1. Upon synthesis from sodium silicate, the surface area and pore volume of porous silica, aluminosilicate, and zinc silicate significantly increased, while the average pore diameter notably decreased. This suggests that bagasse fly ash can be used to produce sodium silicate, enabling the synthesis of silica, aluminosilicate, and zinc silicate with high porosity. Zinc silicates exhibited 1.39–1.82 and 0.68–0.76 times higher average pore diameter but lower surface area than aluminosilicate, respectively.

**Table 1:** Physical properties of synthesized adsorbents.

Sample	BET surface area (m^2^/g)	Average pore diameter (nm)	Pore volume (cm^3^/g)
Sodium silicate	0.92	58.62	0.017
SiO_2_	408.95	7.27	0.74
AS(0.02)	389.51	9.31	0.94
AS(0.04)	439.08	7.48	0.82
AS(0.08)	466.06	5.30	0.62
ZS(0.02)	286.00	13.00	0.93
ZS(0.04)	298.98	11.62	0.87
ZS(0.08)	354.52	9.69	0.86

### Crystalline structure

**Figure 3:**
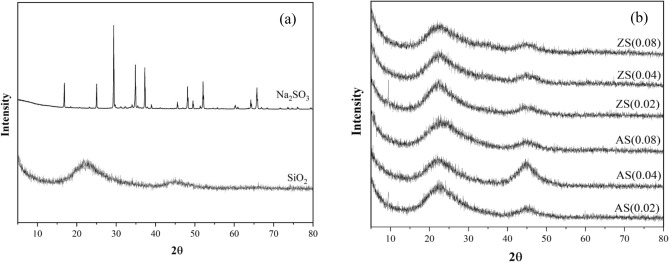
XRD patterns of (**a**) sodium silicate and silica (**b**) aluminosilicate and zinc silicate.

Figure 3a illustrates the crystalline structure of sodium silicate derived from bagasse fly ash after undergoing calcination. The crystalline peaks spanned from 2θ values of 16.84°–65.73°, with the highest intensity peak at 29.38°. Additionally, porous silica exhibited distinct broad peaks at 2θ ranging between 15° and 25°, indicating its amorphous silica nature. In Fig. 3b, the crystalline structure of aluminosilicate and zinc silicate is presented. The crystalline peaks of all samples suggest strong broad peaks within the 2θ range of 15°–30° and 40°–50°. These observations suggest the presence of an amorphous silica structure, notably pronounced in aluminosilicate and zinc silicate samples with a low molar ratio of aluminum or zinc to silicon^28^.

### FTIR spectra

**Figure 4:**
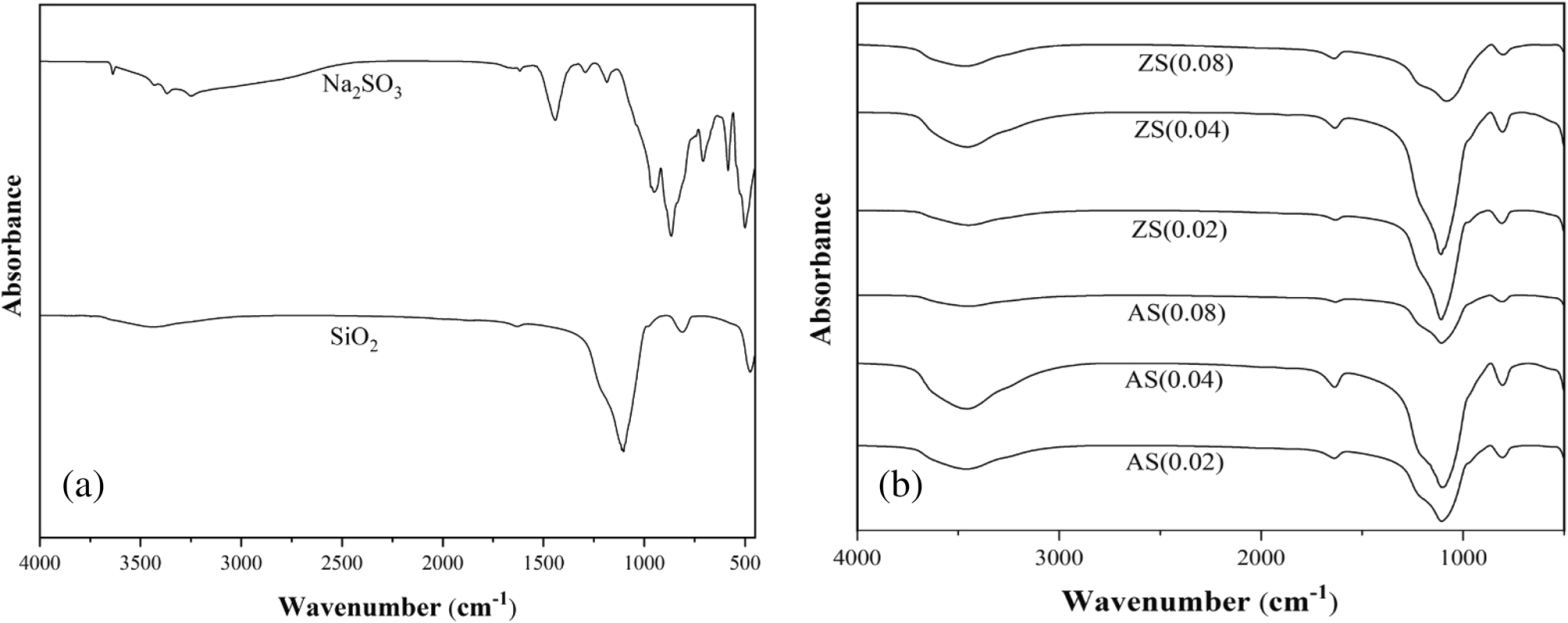
FTIR spectra of (**a**) sodium silicate and silica (**b**) aluminosilicate and zinc silicate.

**Figure 5:**
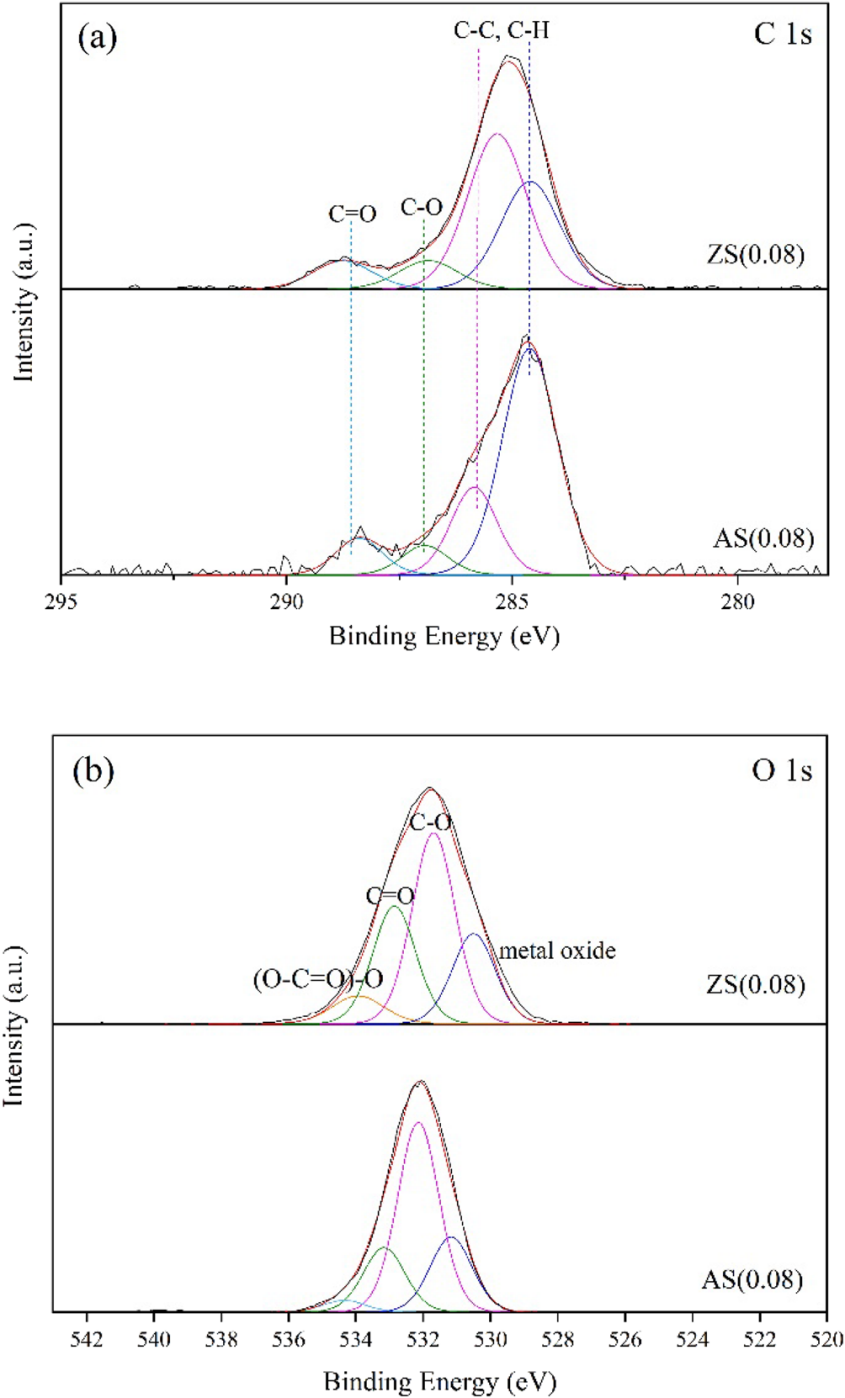
X-ray photoelectron spectroscopy (XPS) spectra of aluminosilicate and zinc silicate (**a**) C 1 s and (**b**) O 1 s.

Figure 4a illustrates the functional groups presented in sodium silicate and porous silica. In sodium silicate, the distinct bands at 3249–3369 cm^−1^ signify the presence of the silanol OH group and adsorbed water, while the broader bands at 951 cm^−1^ corresponds to the Si–O–Si bond group, representing the siloxane group within the isolated structure. In Fig. 4b, all synthesized aluminosilicate and zinc silicate samples exhibited strong peaks within the asymmetry Si–O–Si range of 1080–1110 cm^−1^^29^. These peaks indicate the presence of a tetrahedral network structure of silica with oxygen. Additionally, stretching vibrations of the symmetry Si–O–Si at 805 cm^−1^ and bending vibrations of the symmetry Si–O at 475 cm^−1^ were observed. A broad band at 3450 cm^−1^ and a peak at 1635–1640 cm^−1^ can be attributed to the stretching and bending vibrations of the hydroxyl group (O–H), respectively^30^. Importantly, all synthesized adsorbents demonstrated a distinct Si–O–Si group structure compared to sodium silicate. According to Alexander et al.^31^, an increase in the tetrahedral structure of the Si–O–Si group enhances the adsorption ability of silica-based adsorbents. Notably, porous silica exhibited spectra like those of aluminosilicate and zinc silicate (Fig. 5a).

### XPS

X-ray photoelectron spectroscopy (XPS) spectra of aluminosilicate and zinc silicate are shown in Fig. 5. The spectra of C 1 s and O 1 s are displayed in Fig. 5a,b, respectively. Figure 5a shows the C 1 s spectrum peaks; the first two peaks at around 284.6 and 285.5 eV correspond to C–C and C–H bonds; 286.9 eV corresponds to C–O bonds, and the peak at 288.3–288.7 eV corresponds to C=O (carbonyl) bonds^32^. The O 1 s XPS spectra exhibited four peaks, as shown in Fig. 5b. The peak at around 531 eV corresponds to metal oxides (Si–O–Si, Si–O–Al, and Si–O–Zn), while the peaks at about 532, 532.8, and 533.8 eV correspond to C–O, C=O, and (O–C=O)–O groups, respectively^33,34^.

### Morphology

**Figure 6:**
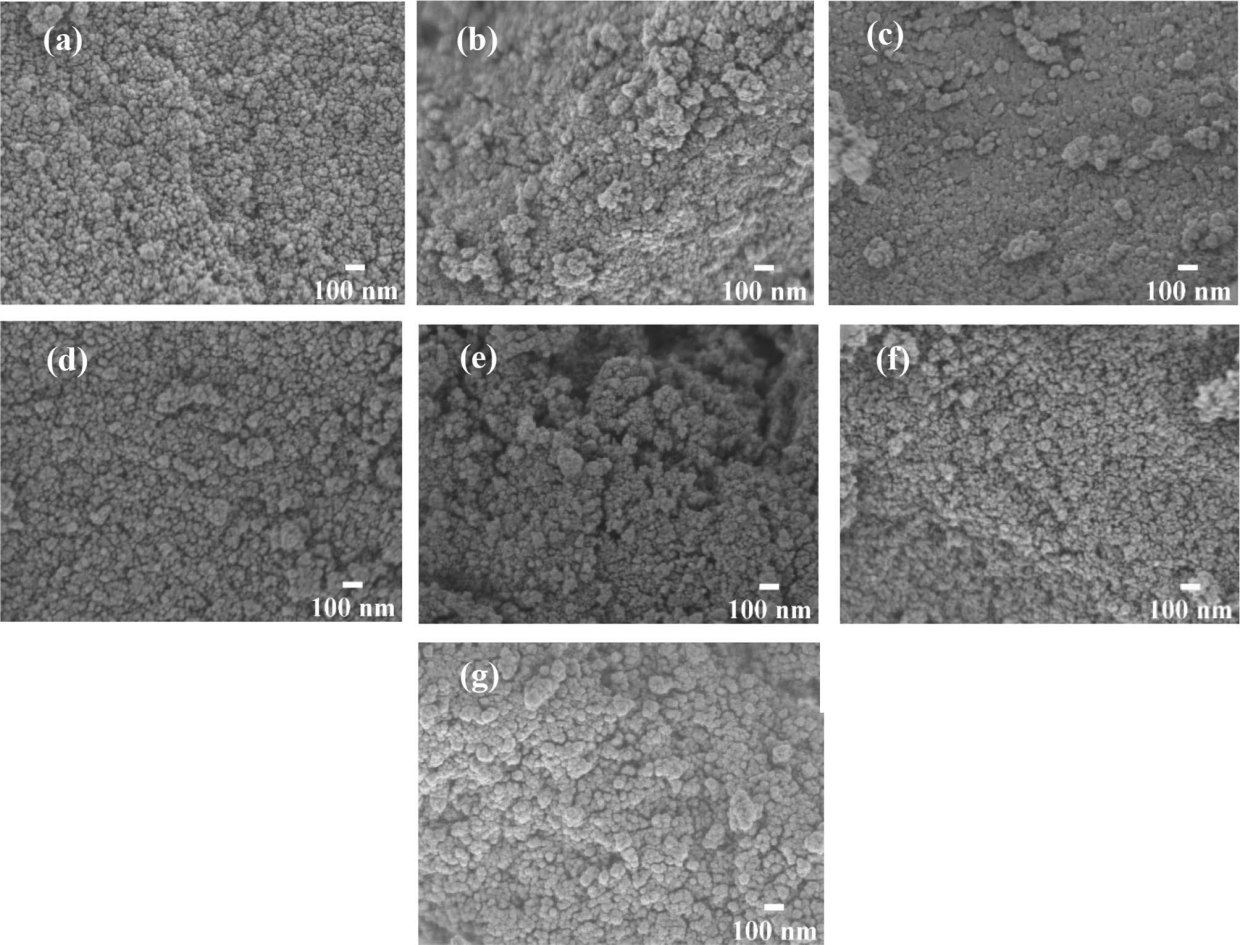
FE-SEM images of (**a**) AS(0.02), (**b**) AS(0.04), (**c**) AS(0.08), (**d**) ZS(0.02), (**e**) ZS(0.04), (**f**) ZS(0.08) and (**g**) Silica (SiO_2_).

Figure 6 illustrates the surface morphology of silica, aluminosilicate, and zinc silicate, portraying spherical particle shapes in all samples. Comparatively, aluminosilicate and zinc silicate exhibited smaller particle sizes than silica, and this decrease in size corresponded with higher molar ratios of aluminum or zinc to silicon. This correlation was consistent with the findings of N_2_ sorption analysis, which indicated a higher surface area but smaller pore size in these samples.

**Figure 7:**
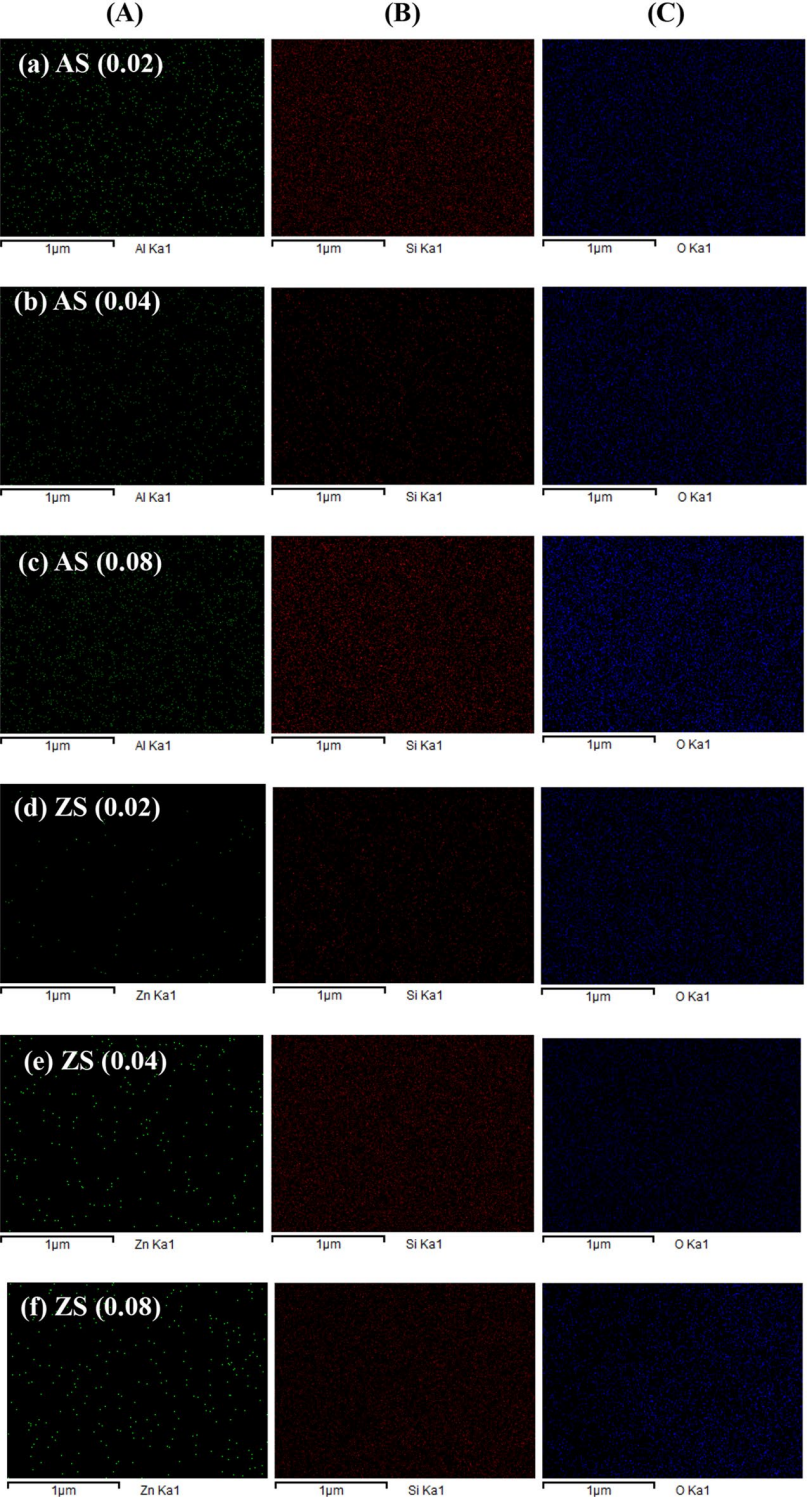
EDS mapping of aluminosilicate and zinc silicate (**A**) Al or Zn (**B**) Si and (C) O.

In Fig. 7, the EDS mapping of aluminosilicate and zinc silicate demonstrates a relatively uniform distribution of Si, Al, Zn, and O atoms across the material surfaces. Specifically, the reported atomic ratios of Al to Si on the surface of aluminosilicates were 0.019, 0.036, and 0.068 for AS(0.02), AS(0.04), and AS(0.08), respectively. Similarly, the atomic ratios of Zn to Si on the surface of zinc silicate were 0.019, 0.039, and 0.072 for ZS(0.02), ZS(0.04), and ZS(0.08), respectively. It can be observed that aluminum exhibited greater uniform dispersion in the same regions of silicon and oxygen throughout the surface compared to zinc. Additionally, the uniformly smaller particle size of aluminosilicate relative to zinc silicate is evident in Fig. 6. This indicates that the aluminosilicate formed a matrix with higher homogeneity.

**Figure 8:**
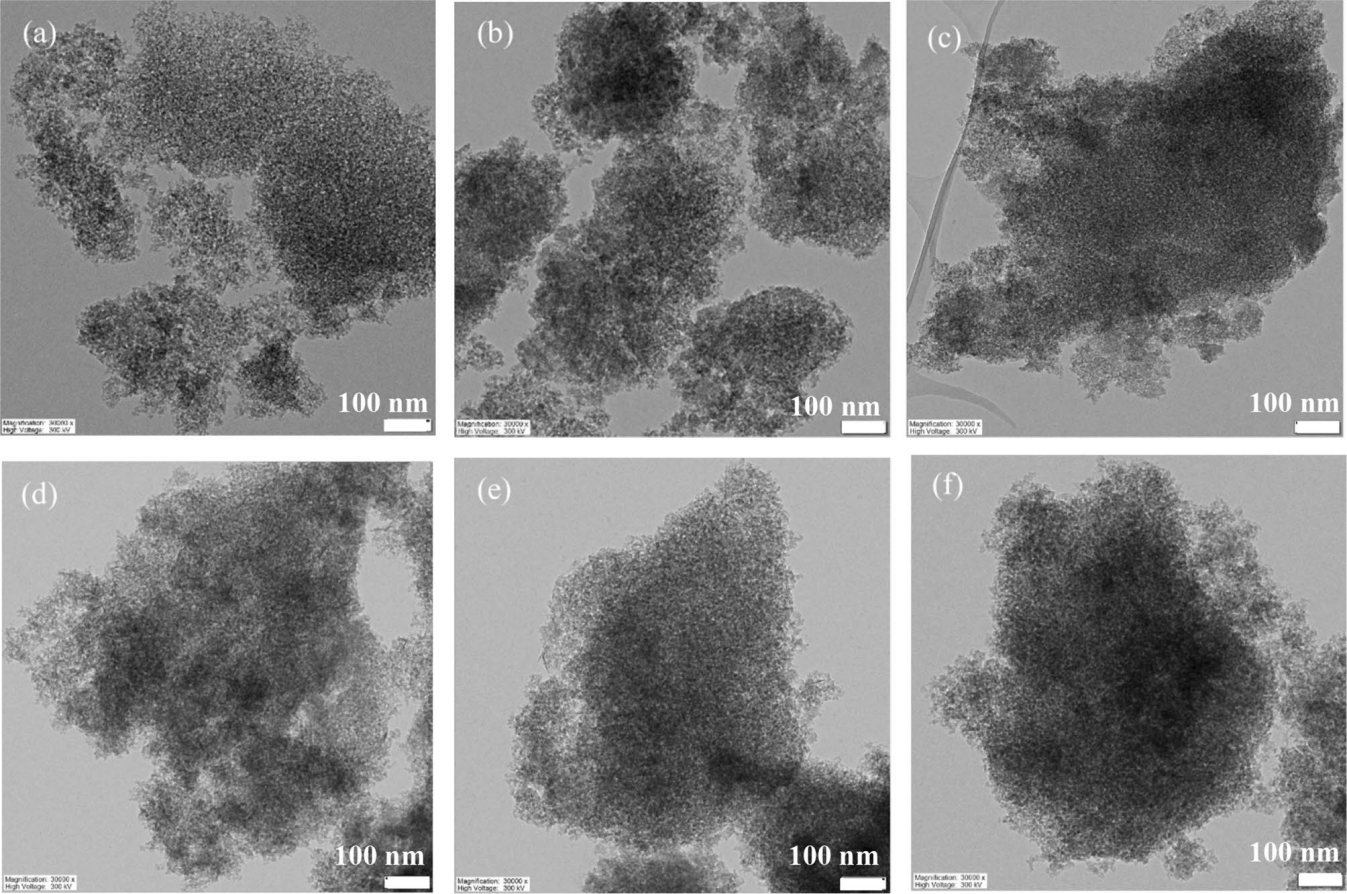
FE-TEM images of (**a**) AS(0.02), (**b**) AS(0.04), (**c**) AS(0.08), (**d**) ZS(0.02) (**e**) ZS(0.04), (**f**) ZS(0.08) at magnification of 30,000×.

Figure 8, which utilizes FE-TEM, validates the uniform distribution of aluminum and zinc in aluminosilicate and zinc silicate, respectively. Samples with higher molar ratios between the metal and silicon showcase a denser dispersion of the metal. This finding suggests a uniform dispersion of aluminum and zinc, both on the surface and within the pores/interparticle channels of aluminosilicate and zinc silicate.

### NH3-TPD profiles

**Figure 9:**
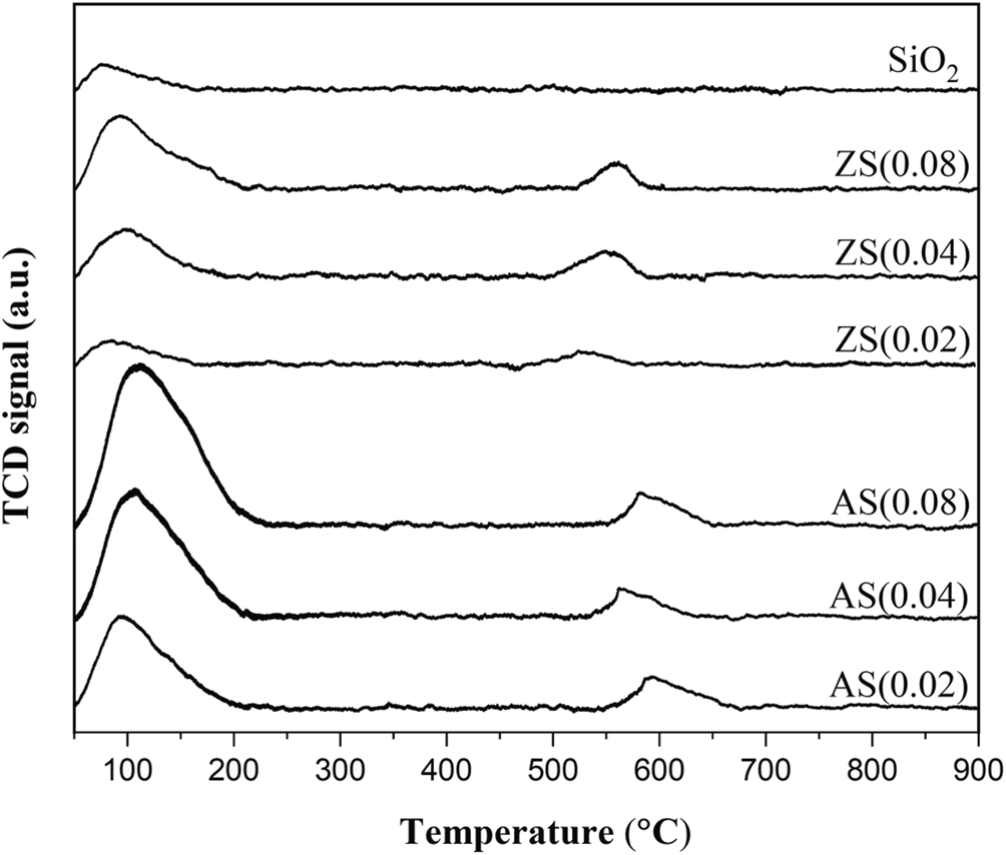
NH_3_-TPD profiles of porous silica, aluminosilicate and zinc silicate.

Figure 9 presents the NH_3_-TPD profiles of the synthesized porous silica, aluminosilicate, and zinc silicate adsorbents. NH_3_ desorption peaks were discerned within two temperature ranges: 100–200 °C, indicating weak acid sites, and 400°–800 °C, reflective of strong acid sites^35^. Porous silica exclusively exhibited intensities associated with weak acid sites, while all aluminosilicate and zinc silicate samples displayed elevated intensities of weak acid sites and subdued intensities of strong acid sites. The intensity of acid sites exhibited an increase with a higher molar ratio of metal to silicon for both aluminosilicate and zinc silicate cases. This increase is attributed to the higher content of aluminum or zinc, which led to a higher level of bonding between these metals and oxygen. The presence of Si–O–Al and Al–O bond groups in aluminosilicate, as well as Si–O–Zn and Zn–O bond groups in zinc silicate, contributed to an increase in the surface acidic region^36,37^. Aluminosilicate demonstrated a superior intensity of acid sites compared to zinc silicate and porous silica, respectively. This is attributed to aluminum’s lower electronegativity compared to zinc, making it more prone to losing electrons. Oxygen exhibits vigorous reactivity with atoms that readily lose electrons, thereby favoring the formation of Si–O–Al and Al–O bond groups in aluminosilicate over Si–O–Zn and Zn–O bond groups in zinc silicate.

### Zeta potentials

Table 2 presents the zeta potentials observed in porous silica, aluminosilicate, and zinc silicate. Zeta potential, defining the electrokinetic potential within colloidal systems, serves as an indicator of surface charge characteristics. Notably, the pH level of the medium exerted influence on the zeta potential, revealing negatively charged surfaces across all samples. Aluminosilicates, notably AS(0.04) and AS(0.08) adsorbents displayed significantly higher levels of negative surface charge compared to zinc silicates and porous silica at both pH 2.5 and 6.5, respectively.

**Table 2:** Zeta potentials of aluminosilicate and zinc silicate.

Sample	Zeta Potential (mV)	
	pH	
	2.5	6.5
SiO_2_	− 18.74	− 31.45
AS(0.02)	− 17.98	− 31.15
AS(0.04)	− 20.52	− 34.14
AS(0.08)	− 22.02	− 36.79
ZS(0.02)	− 12.62	− 27.36
ZS(0.04)	− 14.03	− 30.18
ZS(0.08)	− 16.43	− 32.73

**Scheme 1:**
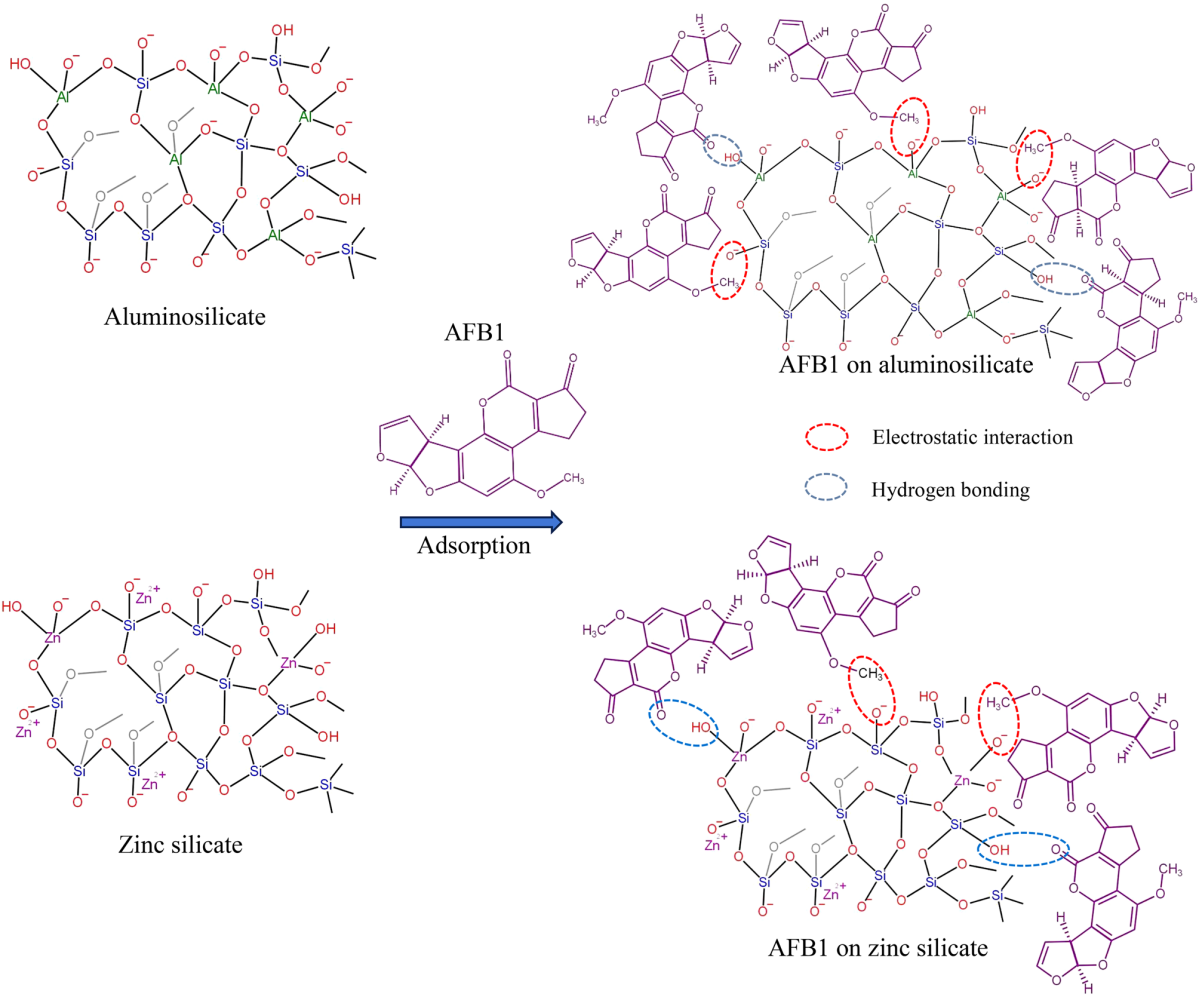
Pictorial tetrahedral structures without long-range order of amorphous aluminosilicate and zinc silicate and plausible adsorption mechanism of AFB1.

Scheme 1 illustrates the possible structures of aluminosilicate and zinc silicate and the adsorption mechanism of AFB1. Aluminosilicate and zinc silicate, in their amorphous forms, may exhibit a tetrahedral network structure with a short range-order^38,39^. The lower intensity of the 1080–1110 cm^−1^ peaks, associated with asymmetric Si–O–Si vibrations in aluminosilicate, implies fewer Si–O–Si bonds in aluminosilicate compared to zinc silicate. Aluminum exhibits a high degree of isomorphic substitution, where Al^3+^ replaces Si^4+^ in the silica network^40^. This substitution introduces a net negative charge because Al^3+^ has a lower charge than Si^4+^. In contrast, zinc has a lower potential for isomorphic substitution and is likely to be associated with complexes in the form of zinc ions (Zn^2+^)^41^. Therefore, the lower negative charge in zinc silicate can be observed as the lower negative values of the zeta potential. The higher surface negative charge of aluminosilicate was expected to increase anion attraction on the adsorbent surface, thereby enhancing the electrostatic interaction with the cationic components of AFB1 molecules^15^.

### In vitro AFB1 adsorption capability

Table 3 presents the in vitro AFB1 adsorption capacities of the synthesized adsorbents, revealing an increase in AFB1 adsorption with extended in vitro digestion times. A noticeable increase in AFB1 adsorption was observed at pH 7 (intestinal phase) compared to pH 2 (gastric phase). This indicates that the main adsorption mechanism of AFB1 on aluminosilicate and zinc silicate involves electrostatic interactions, facilitated by extensive deprotonation of the adsorbents^30^. AFB1 cations adhered to the adsorbents due to electrostatic attraction by anion attraction, thereby balancing the surface charge of the adsorbents^42^. In addition, the FTIR results indicate the presence of hydroxyl group (O–H) on aluminosilicate and zinc silicate. Therefore, hydrogen bonding can occur between these adsorbent and AFB1 molecules. Figure S1 shows the FTIR of aluminosilicate (AS(0.08)) before and after adsorption of AFB1. It was found that after adsorption of AFB1, the aluminosilicate exhibited lower intensity peaks at 3450 cm^−1^ and 1635–1640 cm^−1^, attributed to the stretching and bending vibrations of the hydroxyl group (O–H), respectively. Additionally, the peaks at 951 cm^−1^ and 1080–1110 cm^−1^, corresponding to the Si–O–Si bond group and the asymmetric Si–O–Si range, respectively, also showed reduced intensity compared to those before adsorption. These FTIR results confirmed the adsorption of AFB1 on the aluminosilicate by electrostatic interaction and hydrogen bonding. Scheme 1 presents the mainly plausible adsorption mechanism of AFB1 on the aluminosilicate and zinc silicate. Additionally, the presence of C–C bonds in the XPS results suggests that hydrophobic interactions may have occurred between the carbon atoms of the silicates and those of AFB1.

**Table 3:** In vitro AFB1 adsorption capacities of aluminosilicate and zinc silicate.

Sample	Adsorption (%)	
	Digestion phase	
	After gastric phase	After intestinal phase
SiO_2_	4.02 ± 0.29^a^	49.28 ± 2.74^a^
AS(0.02)	17.43 ± 1.12^bc^	84.76 ± 3.34^d^
AS(0.04)	18.50 ± 1.37^c^	87.59 ± 3.66^de^
AS(0.08)	18.78 ± 1.55^c^	88.25 ± 3.12^e^
ZS(0.02)	15.37 ± 0.92^b^	78.75 ± 2.02^b^
ZS(0.04)	16.19 ± 1.18^b^	80.80 ± 2.87b^c^
ZS(0.08)	16.72 ± 1.04^b^	81.97 ± 2.53^c^

The means in the same column sharing the same superscript letter are not significantly different as determined by Tukey's test (P>0.05).

Aluminosilicate displayed higher AFB1 adsorption levels after 5 h (post-gastric phase) and 7 h (post-intestinal phase) of in vitro digestion compared to zinc silicate. Aluminosilicate exhibited a higher BET surface area, surface acidic site, and negatively charged surface than zinc silicate, thereby accelerating AFB1 adsorption through increased contacting area and electrostatic interactions. The adsorption efficiency increased with a higher molar ratio of aluminum or zinc to silicon, attributable to the augmentation in surface acidic sites and a negatively charged surface. However, the adsorption capacities of samples AS(0.04) and ZS(0.04) did not significantly differ from those of AS(0.08) and ZS(0.8), respectively. This suggests a decline in adsorption ability as the molar ratio of aluminum or zinc to silicon exceeded 0.04. This phenomenon might be associated with the trade-off between a reduction in average pore size and pore volume against an increase in zeta potential value and surface acidic sites of the adsorbents as the aluminum or zinc content increased. The surface properties of AS(0.08) and ZS(0.8) following the adsorption of AFB1 were assessed using N_2_ physisorption analysis. The BET surface area, average pore diameter, and pore volume of AS(0.08) and ZS(0.8) were determined to be 341.78 and 255.13 m^2^/g, 5.69 and 10.59 nm, and 0.48 and 0.67 cm^3^/g, respectively. A notable observation was the considerable decrease in specific surface areas and pore volumes of AS(0.08) and ZS(0.8) after the adsorption of AFB1, in comparison to their original states (Table 1). This decline is consistent with the principle of “pore blockage,” where the intrusion of organic molecules into the pores leads to a reduction in the BET surface area. This phenomenon suggests the occurrence of both surface and interpore adsorption of organic molecules^43^.

### AFB1 in vitro adsorption kinetics

**Figure 10:**
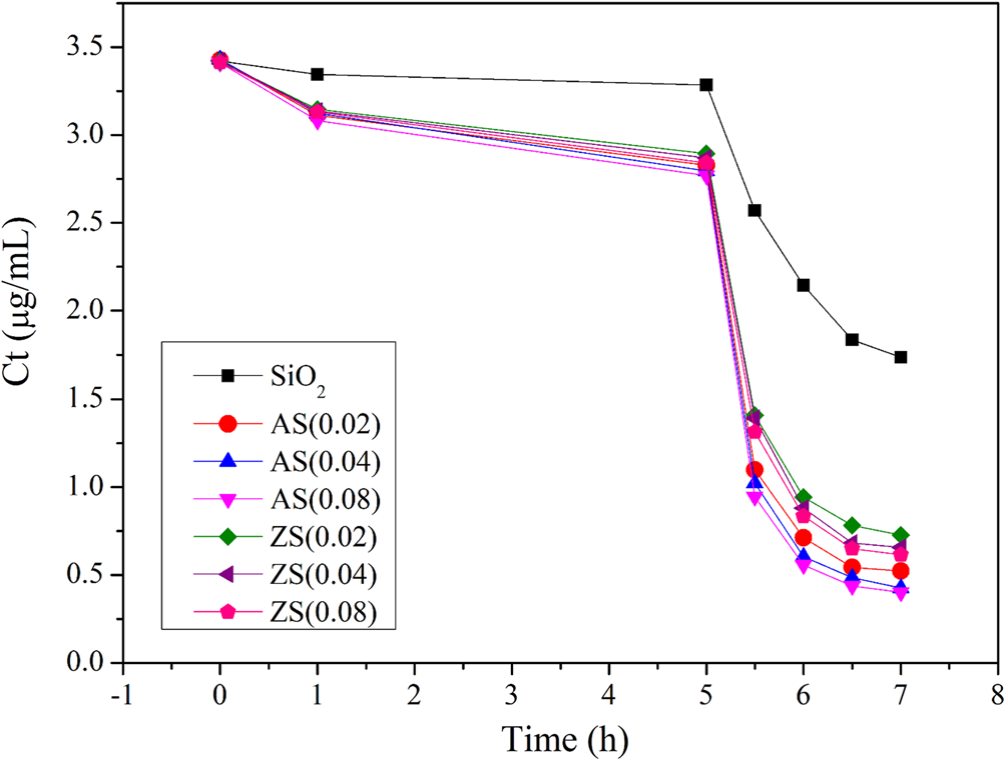
Concentrations of AFB1 during in vitro digestion: gastric phase (0–5 h) and intestinal phase (5–7 h).

Figure 10 presents the changes in AFB1 concentration during in vitro digestion, indicating a limited reduction in AFB1 levels during the gastric phase across all cases. However, higher adsorption rates were evident during the intestinal phase^19^. This observed trend correlates with the notably higher negative charge on the surfaces of silica and bentonite at pH 6.5 compared to pH 2.5^44^.

**Figure 11:**
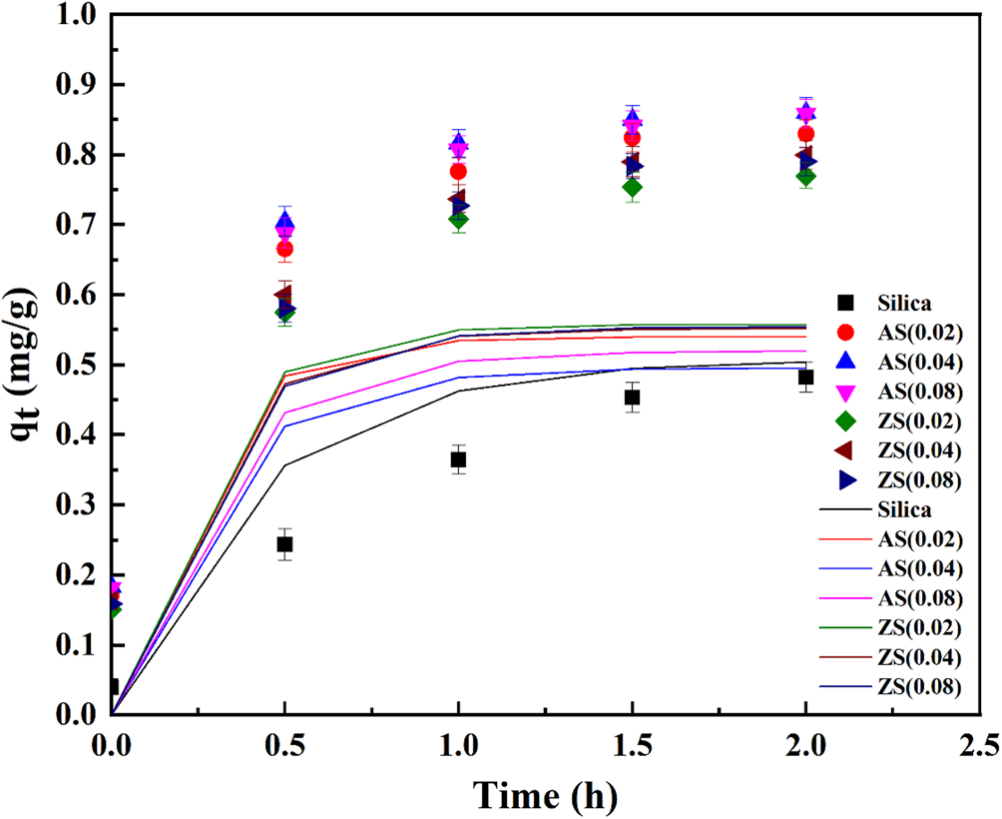
Pseudo-first-order kinetic model results of AFB1 adsorption on synthesized porous silica, aluminosilicate, and zinc silicate.

**Figure 12:**
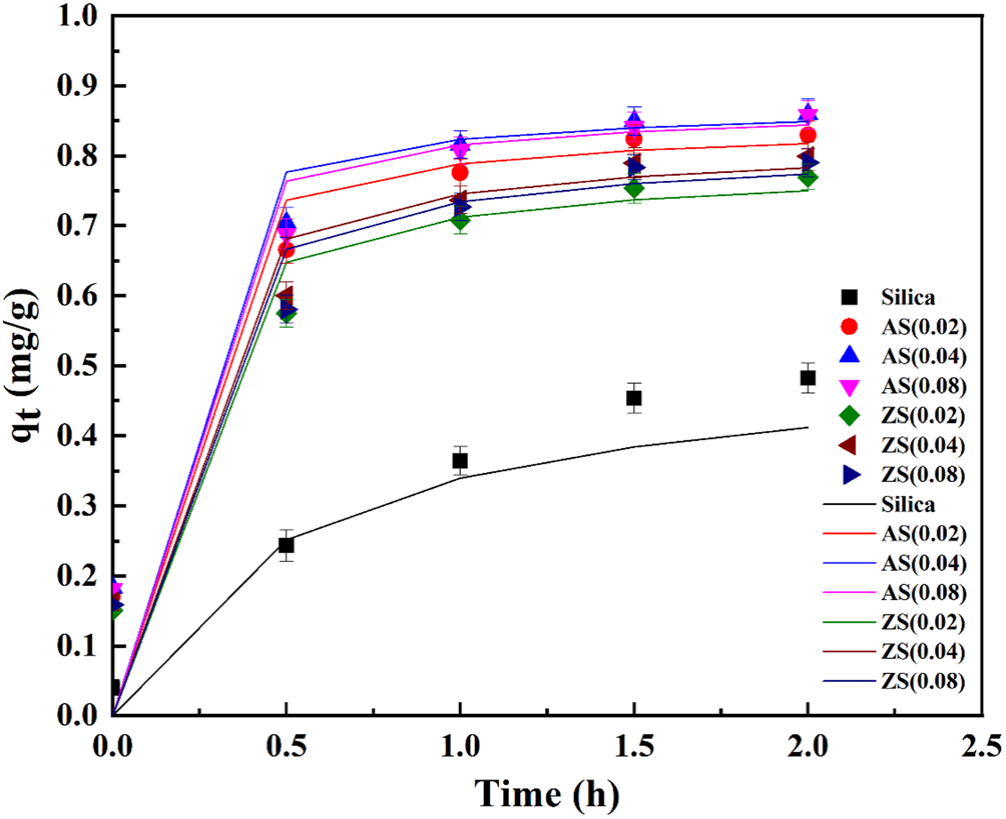
Pseudo-second-order kinetic model results of AFB1 adsorption on synthesized porous silica, aluminosilicate, and zinc silicate.

**Figure 13:**
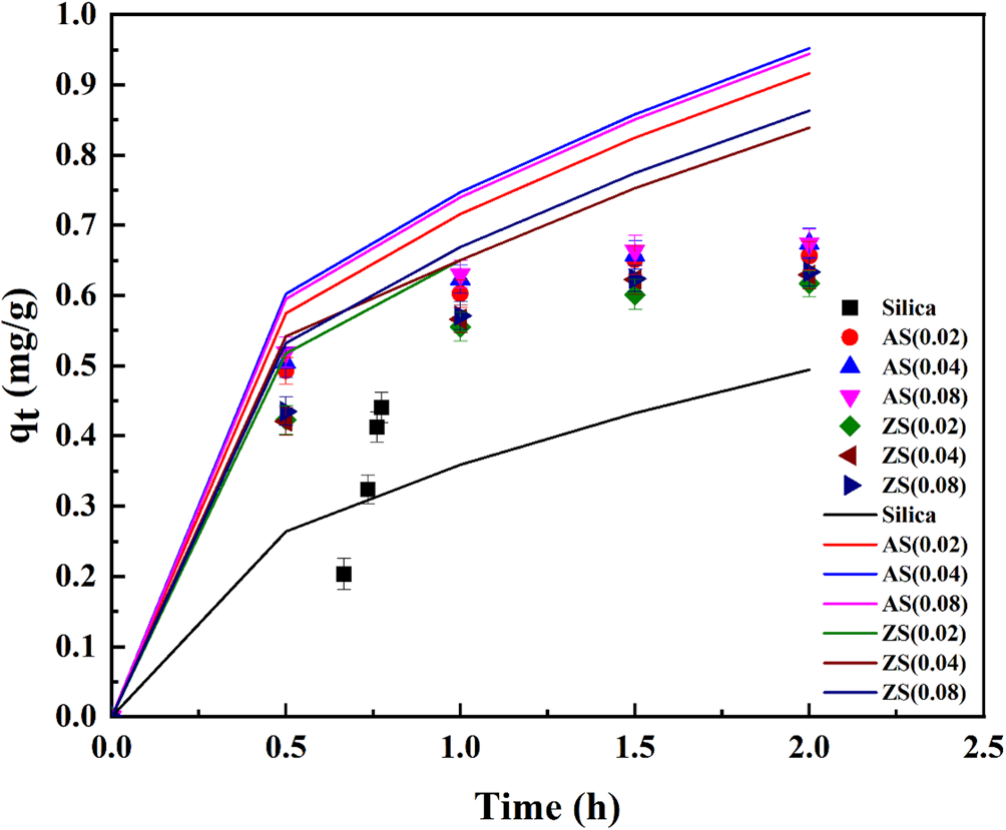
Intra-paricle diffusion model results of AFB1 adsorption on synthesized porous silica, aluminosilicate, and zinc silicate.

Figures 11, 12, and 13 presented the kinetics of AFB1 adsorption on synthesized porous silica, aluminosilicate, and zinc silicate during the in vitro intestinal phase. These kinetics were described by the pseudo-first order, pseudo-second order, and intra-particle diffusion model equations, respectively. The parameters of these kinetic models are detailed in Table 4. Notably, the pseudo-first-order kinetic model best represents the experimental data of porous silica. This indicates that the adsorption rate of AFB1 onto porous silica primarily relied on physisorption. In contrast, AFB1 adsorption on aluminosilicate and zinc silicate is more consistent with the pseudo-second-order model. This implies that the adsorption rate of AFB1 on aluminosilicate and zinc silicate was probably limited by chemisorption, reliant on the adsorption capacity of the adsorbents^45^.

**Table 4:** Parameters of kinetic models for AFB1 adsorption using synthesized adsorbents.

Model parameter	SiO_2_	AS(0.02)	AS(0.04)	AS(0.08)	ZS(0.02)	ZS(0.04)	ZS(0.08)
*q*_e(exp.)_	0.5485	0.8442	0.8912	0.8895	0.7861	0.8240	0.8194
Pseudo-first-order							
*q*_e (cal.)_ (mg g^-1^)	0.5082	0.5399	0.4958	0.5202	0.5578	0.5520	0.5544
*k*_1_ (h^-1^)	2.4094	4.5275	3.5655	3.5330	4.2133	3.8845	3.4761
*R*^2^	0.9939	0.9718	0.9353	0.9522	0.9917	0.9738	0.9648
Pseudo-second-order							
*q*_e (cal.)_ (mg g^-1^)	0.5233	0.8487	0.8761	0.8747	0.7913	0.8235	0.8177
*k*_2_ (g mg^-1^ h^-1^)	6.7566	18.1965	20.3562	18.0046	14.4151	14.1103	13.1666
*R*^2^	0.9246	0.9956	0.9967	0.9959	0.9921	0.9922	0.9907
Intra-particle diffusion							
*k*_p_ (mg g^-1^ h^-0.5^)	0.3255	0.4829	0.4943	0.4936	0.4550	0.4693	0.4684
*C*_i_	0.0336	0.2332	0.2529	0.2460	0.1956	0.2097	0.2007
*R*^2^	0.9917	0.9248	0.9129	0.9232	0.9495	0.9492	0.9542

### Static adsorption isotherms and thermodynamic behavior

Figure S2 shows the impact of the initial concentration of AFB1 on the adsorption capacity of the AS(0.08) sample. The results showed that the higher initial concentration of AFB1 led to a higher decrease in the concentration of AFB1 during the adsorption process. The equilibrium adsorption capacities (*q*_e_) for AFB1 were found to be 1.52 mg/g at 0.3 mg/L, 1.97 mg/g at 0.5 mg/L, 2.03 mg/g at 0.7 mg/L, and 2.25 mg/g at 0.9 mg/L initial concentrations, respectively. The *q*_e_ value increased as the initial AFB1 concentration increased, attributable to the enhanced concentration gradient serving as the driving force for mass transfer. Figure S3 presents the UV–Vis spectra of AFB1 during the static adsorption process. Peaks were observed at wavelengths of 261 nm and 364 nm, consistent with those reported in previous studies^46,47^. It was found that both the peak intensity and the area under the curve decreased with increasing adsorption time, supporting the observed decreasing trend in AFB1 concentration during the adsorption process.

The AFB1 adsorption isotherms for the AS(0.08) sample are illustrated in Figure S4, representing the Langmuir and Freundlich adsorption models. The parameters for these models are provided in Table S1. The adsorption behavior of AFB1 on aluminosilicate aligned more closely with the Langmuir isotherm model, as evidenced by consistently higher *R*^*2*^ values compared to those for the Freundlich model. This suggests that the adsorption of AFB1 onto the aluminosilicate involved a monolayer process on homogenous adsorbent surfaces, which took place at a specific binding site of aluminosilicate^43^.

Figure S5 illustrates the effect of adsorption temperature on the adsorption capacity of AFB1. The experiments were conducted with initial concentrations of 0.5 µg/mL and contact times ranging from 0 to 8 h. The results revealed that the concentration of AFB1 during adsorption was higher at elevated temperatures, indicating a decrease in adsorption capacity with increasing temperature. This phenomenon could be attributed to the aggregation of the porous adsorbent at higher temperatures in an aqueous solution. Such aggregation reduces the available surface area for adsorption, thereby decreasing the total amount of adsorbate that can be adsorbed^48,49^.

The Thermodynamic parameters with temperature in the range of 30–50 °C are reported in Table S2. It is revealed that the negative ∆${\text{G}}^{{{^\circ }}}$ indicates that the adsorption of AFB1 onto AS(0.08) is spontaneous. The calculations found that ∆${\text{H}}^{^\circ }$ values were negative, indicating that the adsorption was exothermic and favored at low temperatures. The results corresponded to the effect of temperature. The positive value of ∆${\text{S}}^{^\circ }$ indicated an increasing disorder and randomness of the molecular motion at the solid–liquid interface during the adsorption process.

Table 5 compares the adsorption capacity (%) for AFB1 among various silicate adsorbents reported in recent studies. This comparison illustrates that aluminosilicate and zinc silicate synthesized from waste sugarcane bagasse fly ash in an amorphous form, prepared with low aluminum/zinc content and at a lower temperature compared to synthesized crystalline silicates, can adsorb AFB1 at levels comparable to several commercial silicates.

**Table 5:** Comparison of adsorption capacity of adsorbents in recent literatures with AS(0.08) and ZS(0.08) for AFB1adsorption.

Adsorbent	Source	Adsorption condition	Adsorbent: AFB1 (w:w)	Adsorption capacity (%)	References
Hydroxyl magnesium silicate (FNHMS)	Commercial sodium silicate	Aqueous Solution, 37 °C AFB1 = 4.0 mg/L FNHMS = 0.02% (w/v)	50:1	82.5	Sun et al.^50^
Hydrated sodium calcium aluminosilicate (HSCAS)	Commercial	In vitro test AFB1 = 0.001 mg/L HSCAS = 0.1% (w/v)	1000000:1	99.98	Yiannikouris et al.^51^
Zeolite	Commercial	In vitro test with poultry diet AFB1 = 0.026 mg/L Zeolite = 0.052% (w/v)	20000:1	81.5	Maguey-González et al.^52^
Smectite clay	Commercial	In vitro test AFB1 = 0.2 mg/L Smectite clay = 0.67% (w/v)	33333:1	99.5	Hernández-Martínez et al.^53^
Zeolite		Zeolite = 0.67% (w/v)		80.97	
Bentonite	Commercial	In vitro test AFB1 = 0.05 mg/L Bentonite = 0.5% (w/v)	100:1	96.59	de Lima Schlösser et al.^54^
*Sugarcane* bagasse fly *ash* (BFA)	*Sugarcane* bagasse fly *ash*	In vitro test AFB1 = 12 mg/L BFA = 0.01% (w/v)	8.33:1	63	de Freitas et al.^45^
AS(0.08)	*Sugarcane* bagasse fly *ash*	In vitro test AFB1 = 0.083 mg/L AS(0.08) = 0.008% (w/v)	1000:1	88.25	This study
ZS(0.08)		ZS(0.08) = 0.008% (w/v)		81.97	

### Reusability

Figure S6 illustrates the adsorption capacity of aluminosilicate (AS(0.08)). The equilibrium adsorption capacity slightly decreases after each regeneration cycle due to some active sites on the aluminosilicate being occupied by AFB1 and not fully recovered through desorption^55^. After four adsorption–desorption cycles, the adsorption capacity showed a significant decrease compared to the initial adsorption process. Therefore, the prepared aluminosilicate could serve as a recyclable adsorbent and be used at least four cycles for the adsorption–desorption of AFB1.

## Conclusion

The synthesized aluminosilicate and zinc silicate derived from sugarcane bagasse fly ash exhibited high specific surface areas and pore volumes. Both materials displayed amorphous structures with a high density of surface acidic sites, particularly weak acidic sites, and a negatively charged surface. Notably, aluminosilicate demonstrated a higher surface area, a greater number of surface acidic sites, and a more negatively charged surface than zinc silicate, resulting in superior adsorption capacity for AFB1. The adsorption of AFB1 onto all adsorbents primarily occurred via electrostatic interactions and hydrogen bonding. Among the synthesized materials, aluminosilicate with an aluminum-to-silicon molar ratio of 0.08 (AS(0.08)) achieved the highest in vitro adsorption efficiency. The pseudo-second-order model best described the adsorption kinetics, indicating that the process was governed by chemisorption. AS(0.08) demonstrated an equilibrium adsorption capacity of 2.25 mg/g at an initial AFB1 concentration of 0.9 µg/mL in an aqueous solution. The adsorption behavior conformed to the Langmuir isotherm model, indicative of monolayer adsorption. Thermodynamic studies suggested that the adsorption process was exothermic and spontaneous. Additionally, this adsorbent exhibited stability, maintaining consistent efficiency over at least four adsorption–desorption cycles.

### Supplementary Information

Supplementary Information.

## Data Availability

The datasets used and/or analyzed during the current study available from the corresponding author on reasonable request.
