# Supplementary material for: Synthesis and characterization of aluminosilicate and zinc silicate from sugarcane bagasse fly ash for adsorption of aflatoxin B1
Source: Sci Rep. 2024 Jun 24;14:14562. doi: 10.1038/s41598-024-65158-2 (PMC11196643; doi:10.1038/s41598-024-65158-2)
Supplement: Supplementary file 1 — Supplementary Information. [file 41598_2024_65158_MOESM1_ESM.docx]

**Supplementary Information**

**Synthesis and characterization of aluminosilicate and zinc silicate from sugarcane bagasse fly ash for adsorption of aflatoxin B1**

**Chalida Niamnuy^a,b^*, Sirada Sungsinchai^c^, Prapaporn Jarernsamrit^a^, Sakamon Devahastin^d,e^, Metta Chareonpanich^a,b^**

a) Department of Chemical Engineering, Faculty of Engineering, Kasetsart University, 50 Ngam Wong Wan Road, Chatuchak, Bangkok 10900, Thailand

b) Center for Advanced Studies in Nanotechnology and Its Applications in Chemical, Food and Agricultural Industries, Kasetsart University, 50 Ngam Wong Wan Road, Chatuchak, Bangkok 10900, Thailand

c) School of Food Industry, King Mongkut’s Institute of Technology Ladkrabang, Bangkok 10520, Thailand

d) Advanced Food Processing Research Laboratory, Department of Food Engineering, Faculty of Engineering, King Mongkut’s University of Technology Thonburi, 126 Pracha u-tid Road, Tungkru, Bangkok 10140, Thailand

e) The Academy of Science, The Royal Society of Thailand, Dusit, Bangkok 10300, Thailand

*Corresponding author.

Tel.: +66 2 797 0999 Ext. 1244; Fax: +66 2 561 4621; E-mail: [fengcdni@ku.ac.th](mailto:fengcdni@ku.ac.th)

**List of Tables**

**Table S1** Estimated isotherm parameters for the static adsorption of AFB1 on aluminosilicate (AS(0.08)).

**Table S2** Thermodynamic parameters for the static adsorption of AFB1 on aluminosilicate (AS(0.08)).

|  | Langmuir isotherm | | |  | Freundlich isotherm | | |
| --- | --- | --- | --- | --- | --- | --- | --- |
|  | *q*_m_  (mg/g) | *b*  _(_L/mg) | *R^2^* |  | *K*_F_  ((mg/g)(L/mg)^1/n^) | *1/n* | *R*^2^ |
|  | 2.5733 | 8.5407 | 0.9942 |  | 2.4558 | 0.2563 | 0.9439 |

**Table S1** Estimated isotherm parameters for the static adsorption of AFB1 on aluminosilicate (AS(0.08)).

**Table S2** Thermodynamic parameters for the static adsorption of AFB1 on aluminosilicate (AS(0.08)).

| T  (K) | *K*_D_ | Δ*G*^°^  (kJ mol^-1^) | Δ*H*^°^  (kJ mol^-1^) | Δ*S*^°^  (J K^-1^mol^-1^ ) |
| --- | --- | --- | --- | --- |
| 303 | 5.89 | -4.47 | -2.16 | 7.00 |
| 313 | 5.83 | -4.44 |  |  |
| 323 | 5.16 | -4.14 |  |  |

**List of Figures**

**Figure S1** FTIR spectra of aluminosilicate (AS(0.08)) (a) before in vitro adsorption (b) after in vitro adsorption.

**Figure S2** Concentration of AFB1 during static adsorption on aluminosilicate (AS(0.08)) at various initial concentrations.

**Figure S3** Spectra for AFB1 during static adsorption on aluminosilicate (AS(0.08)) at an initial concentration of 0.5 µg/mL.

**Figure S4** Isotherms of AFB1 static adsorption on aluminosilicate (AS(0.08)).

**Figure S5** Concentration of AFB1 during static adsorption on aluminosilicate (AS(0.08)) at various temperatures.

**Figure 6** Adsorption capacities of AFB1 on aluminosilicate (AS(0.08)) at various numbers of adsorption-desorption cycle.

**Figure S1** FTIR spectra of aluminosilicate (AS(0.08)) (a) before in vitro adsorption (b) after in vitro adsorption.

**Figure S2** Concentration of AFB1 during static adsorption on aluminosilicate (AS(0.08)) at various initial concentrations.

**Figure S3** Spectra for AFB1 during static adsorption on aluminosilicate (AS(0.08)) at an initial concentration of 0.5 µg/mL.


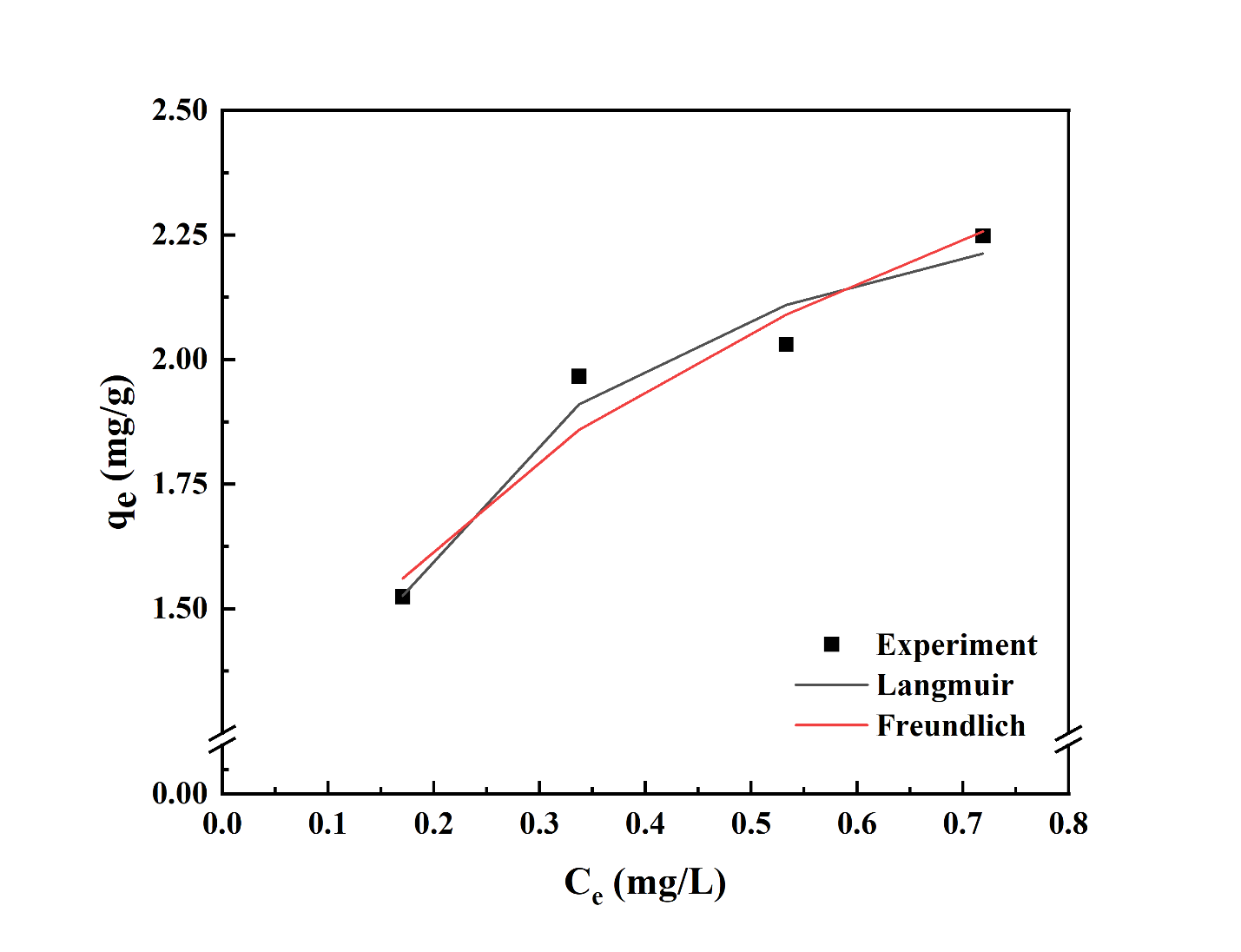


**Figure S4** Isotherms of AFB1 static adsorption on aluminosilicate (AS(0.08)).

**Figure S5** Concentration of AFB1 during static adsorption on aluminosilicate (AS(0.08)) at various temperatures.

a

a

ab

ab

b

**Figure S6** Adsorption capacities of AFB1 on aluminosilicate (AS(0.08)) at various numbers of adsorption-desorption cycle. The different letters are statistically different values (*p* ≤ 0.05).
